# The Effect of Nursing Students' Self-Efficacy on Patient-Centered Communication During the COVID-19 Pandemic: The Mediating Effect of Learning Burnout

**DOI:** 10.3389/fpsyt.2021.787819

**Published:** 2021-12-02

**Authors:** Jing Wang, Qiuyue Zheng, Wei Song, Ling Wei

**Affiliations:** ^1^Department of Applied Psychology, School of Health, Fujian Medical University, Fuzhou, China; ^2^Student Affair Department, Fujian Health College, Fuzhou, China

**Keywords:** COVID-19, nursing students, general self-efficacy, learning burnout, patient-centered communication

## Abstract

**Background:** The 2019 coronavirus disease (COVID-19) outbreak has put the global health system under the spotlight. As part of the medical workforce, nurses play an important role in interacting with and caring for patients; hence, patient-centered communication (PCC) has been emphasized in nursing education. Thus, it is worth investigating how future nurses perceive PCC and PCC-related factors under the special circumstances of COVID-19. For this purpose, the present study analyzed the mechanisms underlying the association between self-efficacy and nurse–patient communication tendency through learning burnout among nursing students during the COVID-19 pandemic.

**Methods:** The general self-efficacy questionnaire, college students' learning burnout scale, and doctor–patient communication tendency scale were used to survey 2,231 nursing students in higher vocational medical colleges at the onset of the COVID-19 pandemic.

**Results:** General self-efficacy can directly negatively correlate with the degree of nursing students' overall nurse–patient communication, including caring, sharing, and health promotion. Dejection from learning burnout partially mediated the relationships between self-efficacy and caring and between self-efficacy and sharing; it fully mediated the relationship between self-efficacy and health promotion. Reduced personal accomplishment partially mediated between self-efficacy and caring, while it fully mediated between self-efficacy and health promotion; however, it did not play a role in the sharing model.

**Conclusion:** Self-efficacy influences nurse–patient communication through learning burnout. Specifically, dejection and reduced personal accomplishment—two aspects of learning burnout—may compromise nursing students' willingness to engage in PCC. Thus, the importance of PCC, especially during critical health situations such as pandemics, should be emphasized further in future nursing education.

## Introduction

On March 11, 2020, the World Health Organization declared the outbreak of the 2019 coronavirus disease (COVID-19) a pandemic (1). This highly infectious disease remains a global health risk to date. To fight such a disease, biological, psychological, and social factors must be integrated into the patient recovery process. However, under these stressful circumstances, nursing staff must manage heavy workloads, long hospital hours, and fears of contagion as well as overcome the difficulties of communicating through layers of personal protection equipment. These difficulties may impair communication with patients, causing health workers to focus less on the patients' psychosocial well-being (2). Studies have reported that the quality of perceived nurse–patient communication has decreased since the onset of the COVID-19 pandemic, specifically when discussing treatment and medical procedures (3). To ensure future nurses' readiness to adopt a humanistic approach in medical settings, we must pay attention to how well nursing students perceive nurse–patient communication in a public health crisis such as COVID-19 and identify the factors influencing it.

In the past decades, nurse–patient communication in China was described as patriarchal and characterized by health-worker-centered communication (2). Patients expected health workers to be the experts and tell them what to do (4). Nevertheless, in recent years, most healthcare-related education has emphasized the importance of patient-centered communication (PCC), as it ensures balance and mutual understanding between nurses and patients (5). At the same time, it enhances patient compliance and improves patient satisfaction and health status (6–9).

PCC is a type of nurse–patient communication proposed by Balint in the 1960s. It contrasts with the “illness-oriented medicine” approach, which supports the biological method in patient care (10). The nursing objective of PCC is to strive for individualized care for patients to meet their physiological and psychological needs (11, 12). Epstein et al. (13) described the following PCC features: understanding patients' needs from their perspective and unique psychosocial backgrounds, being respectful and consistent with the patient's values, and sharing an expert understanding of the problem and treatment with them. However, some empirical studies have indicated the gaps between actual and idealized nurse–patient communication, suggesting that the actual nursing condition was often instrumental and focused on procedure rather than personalized patient-centered assessment (10). Therefore, the present study aims to clarify the factors influencing nurse–patient communication tendency in nursing education, specifically, self-efficacy and learning burnout.

Self-efficacy is an individual's confidence in their ability to accomplish certain goals (14). A recent study found that college nursing students' general self-efficacy was positively correlated with their communication ability (15). Similar conclusions were verified for practicing nurses, confirming that the higher their self-efficacy level, the better their clinical communication ability (16, 17). Another study showed that intern nurses who underwent self-efficacy training had greater nurse–patient communication ability than those who did not (18). Thus, in this study, we considered that self-efficacy may play a significant role in patient-centered communication in nursing education.

Learning burnout is a concept derived from job burnout. It refers to students' negative attitude and behavior toward learning caused by learning pressure or lack of interest (19). Learning burnout induces students' feelings of dejection—a sense of frustration or exhaustion due to lack of accomplishment and competence in dealing with academic tasks—which leads to improper behavior, such as skipping classes (19). Learning burnout negatively influences students' academic performance, interpersonal communication, and mental health (20). College students' learning burnout is influenced by both individual and environmental factors, self-efficacy being one of the major individual factors (21, 22). Many empirical findings have shown a negative correlation between self-efficacy and learning burnout among various student groups (23, 24). Nursing students' learning burnout mainly manifests in feelings of depression and improper use of learning strategies (20) and can negatively predict academic burnout (25). Furthermore, the COVID-19 situation could be considered an environmental factor of learning burnout. Indeed, COVID-19 negatively affects medical students' mental health and study performance in general, resulting in increased anxiety and stress (26, 27). Moreover, home quarantine, postponed return to college, and online learning mean that nursing students might be more vulnerable to learning burnout.

Meanwhile, learning burnout may impact nursing students' attitudes toward nurse–patient communication. One study by Williams et al. (28) established a doctor–patient cycle model and recorded how ineffectively managing stress and burnout could lead to a vicious cycle in the medical workplace. Leaving job burnout unaddressed could negatively impact the quality of medical contact with patients, for example, by exhibiting dehumanized behaviors. In this case, health workers might treat patients as operation objects rather than real people (29). Passalacqua and Segrin (12) found that the higher resident physicians' perceived burnout, the worst their patient-centered communication, and the higher their job burnout, the weaker their communication ability (30). Although nursing students have not yet experienced job burnout because they are yet to join the workforce, they are no strangers to learning burnout (31). Therefore, it is worthwhile to explore whether learning burnout could have the same negative relationship to their attitude toward nurse–patient communication, possibly in an indirect and mediating way.

**Figure 1:**
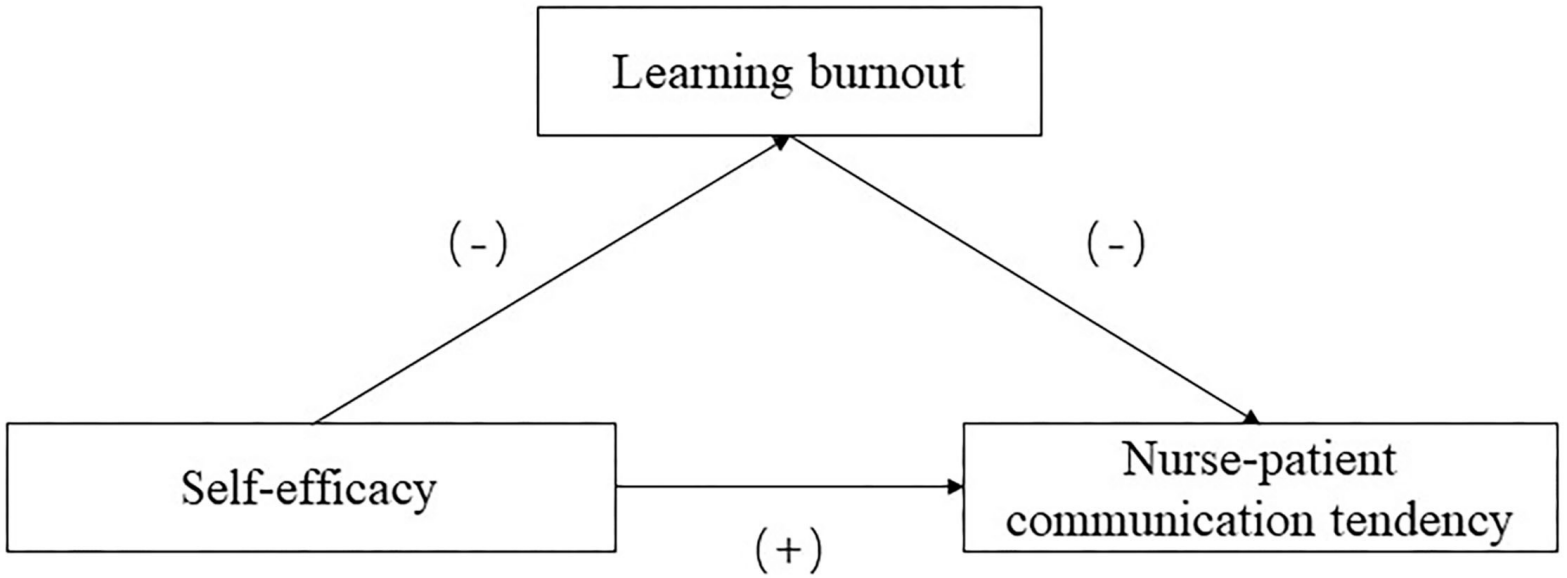
Conceptual model.

This study aimed to explore the mechanisms of the relationship between self-efficacy and nurse–patient communication tendency among nursing students during the COVID-19 pandemic and further uncover the mediating role of learning burnout therein (see Figure 1). By constructing a theoretical model to explain nurse–patient communication tendencies, we sought to provide more insight into the formation of good PCC in future nurses. Given the evidence from previous studies, we hypothesized as follows:

*H1: Self-efficacy positively predicts nurse–patient communication tendency; that is, the higher the self-efficacy is, the stronger the PCC tendency will be, and vice versa*.
*H2: Self-efficacy negatively predicts learning burnout; that is, the higher the self-efficacy is, the lower the learning burnout will be, and vice versa*.
*H3: Learning burnout mediates the relationship between self-efficacy and nurse–patient communication tendency*.

## Materials and Methods

### Participants

For this study, 2,272 nursing students from a higher vocational medical college in Fujian Province were recruited. Forty-one participants were excluded due to incomplete questionnaires. The remaining 2,231 questionnaires, 119 of which were from male participants and 2,112 from female participants, were valid for data analysis. This sample consisted of 705 freshmen, 755 sophomores, and 771 juniors with a mean age of 20.49 years (*SD* = 1.51).

### Procedure

The survey was distributed to the nursing students in February 2020, when the COVID-19 pandemic began to unfold in China. In compliance with the quarantine requirements, we conducted the survey online via a Chinese survey website (www.wjx.cn). All participants completed the survey voluntarily and anonymously.

### Measures

#### General Self-Efficacy Scale

The General Self-Efficacy Scale was developed by Schwarzer and colleagues in 1981 (32) and has been proven to have good reliability and validity when applied to the Chinese college population (33). The scale measures an individual's state of self-efficacy, showing that the higher their score, the better their perceived sense of self-efficacy. Here, participants are asked to rate 10 questionnaire items based on a seven-point Likert scale ranging from 1 (strongly agree) to 7 (strongly disagree). Sample items include “I am confident that I can deal with any unexpected circumstance effectively.” The present sample had good internal consistency (Cronbach's α= 0.90, KMO = 0.93).

#### College Students' Learning Burnout Scale

The College Students' Learning Burnout Scale was revised by Lian et al. (19) according to Maslach's Job Burnout Scale. It is a 20-item scale, measuring three dimensions of learning burnout: dejection, improper behavior, and reduced personal accomplishment. Dejection reveals college students' signs of depressive emotion, lack of interest, and difficulties in managing learning problems. Improper behavior reveals students' inappropriate behaviors associated with learning burnout, such as skipping class, being late to class, and failing to hand in assignments. Reduced personal accomplishment refers to students' low sense of achievement in the learning process due to an inability to complete tasks. Each item is rated on a five-point scale ranging from 1 (strongly disagree) to 5 (strongly agree), whereby the higher the score, the higher the students' negative attitude toward learning. The present sample had good internal consistency (Cronbach's α= 0.88, KMO = 0.92).

#### Doctor–Patient Communication Tendency Scale (DPCTS)

The Doctor–Patient Communication Tendency Scale is a modified version of the classic Patient–Practitioner Orientation Scale by Krupat et al. (34) that assesses communication tendency in the context of the Chinese medical environment (35). In Chinese, doctor–patient communication refers to the communication between patients and medical practitioners in general, including doctors, nurses, and other related health workers (36). This definition was also emphasized at the beginning of the survey. The Doctor–Patient Communication Tendency Scale is a 15-item scale consisting of three dimensions: caring, sharing, and health promotion. The caring dimension is used to measure the extent to which respondents value warmth and support in the doctor–patient relationship and the extent to which medical staff pay attention to psychosocial problems. Sharing measures the extent to which respondents believe that patients are entitled to the same status and power as medical workers and the extent to which they share information with patients. Health promotion measures the respondent's recognition of personalized diagnosis and treatment methods and whether the patient's health should be maintained from a holistic perspective. Health promotion was rated from 1 (totally disagree) to 6 (totally agree). The higher the score, the likelier it is that respondents pay attention to health promotion. Higher scores for caring and sharing indicate patient-centered communication, whereas lower scores represent illness-centered communication. The present sample had good internal consistency (Cronbach's α = 0.76, KMO = 0.89).

### Statistical Analysis

Data was collected using a questionnaire survey. Thus, common method biases were examined first. SPSS 24.0 was used to calculate descriptive statistics and correlations of the study variables. The structural equation model was established using Mplus 8.3 software.

## Results

Harman's single-factor analysis showed that the first factor in our data explained only 19.94% of the variance—less than the critical value (40%)—suggesting that common method bias was unlikely to confound the interpretations of our results (37).

The average score for general self-efficacy was 2.74, slightly above the median level (Median = 2.5), while the results of learning burnout showed that the averages of improper behavior, dejection, and reduced personal accomplishment were below the median level (Median = 3). The results of nurse–patient communication tendency showed that the health promotion and care scores were above the median level, while those of sharing were below it (Median = 3.5). An overview of the correlation coefficients between all variables is presented in Table 1.

**Table 1:** Descriptive statistics and inter-correlations between variables (*n* = 2,231).

**Variable**	**1**	**2**	**3**	**4**	**5**	**6**	**7**
1. General self-efficacy	1						
2. Dejection	−0.29^**^	1					
3. Improper behavior	−0.37^**^	0.67^**^	1				
4. Reduced personal accomplishment	−0.54^**^	0.47^**^	0.50^**^	1			
5. Caring	−0.05^*^	−0.25^**^	−0.15^**^	−0.11^**^	1		
6. Sharing	−0.10^**^	−0.06^**^	−0.02	0.06^**^	0.33^**^	1	
7. Health Promotion	0.19^**^	−0.18^**^	−0.14^**^	−0.21^**^	0.25^**^	−0.33^**^	1
*M*	2.74	2.67	2.82	2.65	4.67	3.44	4.82
*SD*	0.41	0.68	0.61	0.59	0.89	0.90	0.96

* *p < 0.05 (two-tailed)*.

** *p < 0.01 (two-tailed)*.

### Testing for the Mediation Model

To understand how self-efficacy and learning burnout influence nurse–patient communication tendency, mediation analysis was performed for three separate dimensions (sharing, caring, and health promotion) after controlling the sociodemographic variables such as gender and grades. For each mediation analysis, 5,000 bootstrap samples were created to establish a 95% bias-corrected confidence interval for the expected indirect associations.

#### Mediation Effect of Learning Burnout Between Self-Efficacy and Caring

**Figure 2:**
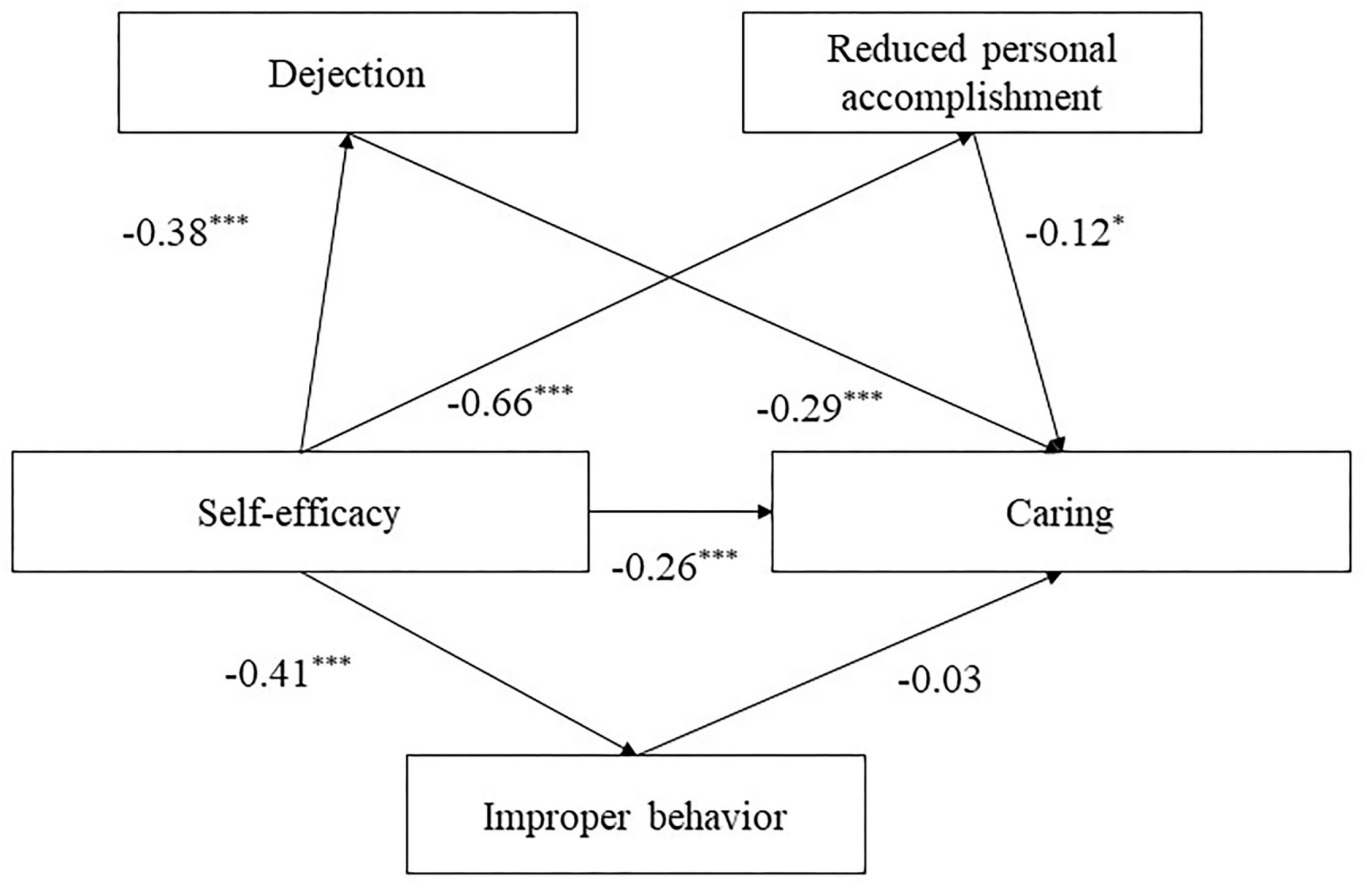
The structural model of self-efficacy, dejection, reduced personal accomplishment and improper behavior on caring. Significant paths are presented by asterisks (*p < 0.05, **p < 0.01, ***p < 0.001).

The results showed that self-efficacy had a significant direct predictive effect on dejection (β = −0.38, *t* = −12.75, *P* < 0.001), improper behavior (β = −0.41, *t* = −12.73, *P* < 0.001), reduced personal accomplishment (β = −0.66, *t* = −33.13, *P* < 0.001), and caring (β = −0.26, *t* = −5.57, *P* < 0.001; see Figure 2). Dejection (β = −0.29, *t* = −7.81, *P* < 0.001) and reduced personal accomplishment (β = −0.12, *t* = −2.54*, p* < 0.05) had a direct predictive effect on caring. Self-efficacy indirectly predicted caring through the mediating effect of dejection (β = 0.11, *t* = 6.66, *P* < 0.001, 95% CI [0.08, 0.14]) and reduced personal accomplishment (β = 0.08, *t* = 2.53, *P* < 0.05, 95% CI [0.02, 0.15]). Self-efficacy had a significant direct effect on caring (β = −0.26, *t* = −5.57, *P* < 0.001, 95% CI [−0.34, −0.17]). However, the total effect of this model was not significant (β = −0.05, *t* = −1.55, *P* > 0.05, 95% CI [−0.10, 0.01]).

#### Mediation Effect of Learning Burnout Between Self-Efficacy and Sharing

**Figure 3:**
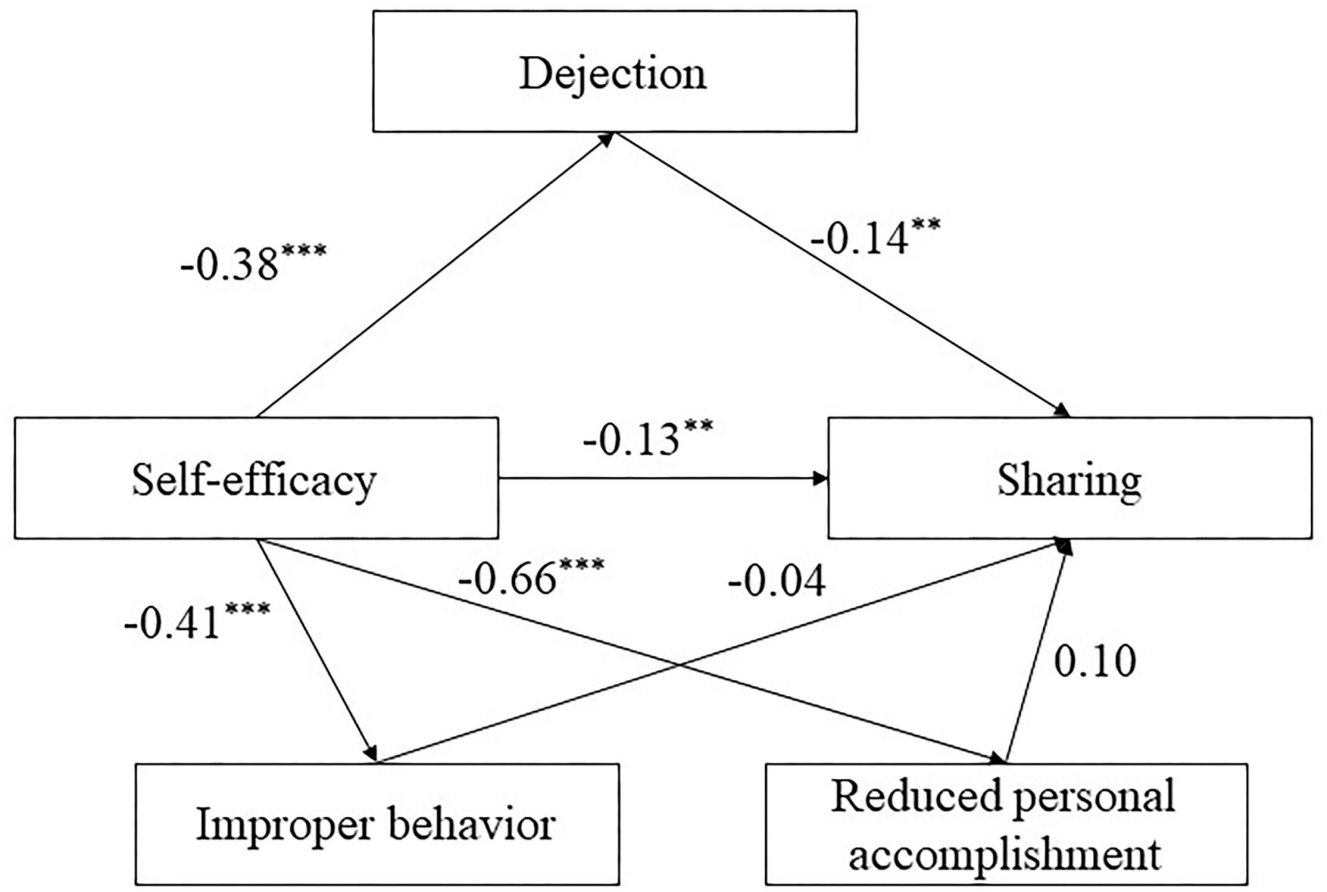
The structural model of self-efficacy, dejection, reduced personal accomplishment and improper behavior on sharing. Significant paths are presented by asterisks (**p < 0.01, ***p < 0.001).

Self-efficacy had a significant direct effect on dejection (β = −0.38, *t* = −12.75, *P* < 0.001), improper behavior (β = −0.41, *t* = −12.73, *P* < 0.001), reduced personal accomplishment (β = −0.66, *t* = −33.13, *P* < 0.001), and sharing (β = −0.13, *t* = −2.68, *P* < 0.01; see Figure 3). Dejection had a significant direct effect on sharing (β = −0.14, *t* = −3.42, *P* < 0.01). Self-efficacy showed a significant direct effect on sharing (β = −0.13, *t* = − 2.68, *P* < 0.01, 95% CI [−0.23, −0.03]) and a significant indirect effect on sharing through the mediation of dejection (β = 0.05, *t* = 3.40, *P* < 0.01, 95% CI [0.02, 0.08]). The total effect of this mediation model was significant (β = −0.13, *t* = −3.80, *P* < 0.001, 95% CI [−0.19, −0.06]).

#### Mediation Effect of Learning Burnout Between Self-Efficacy and Health Promotion

**Figure 4:**
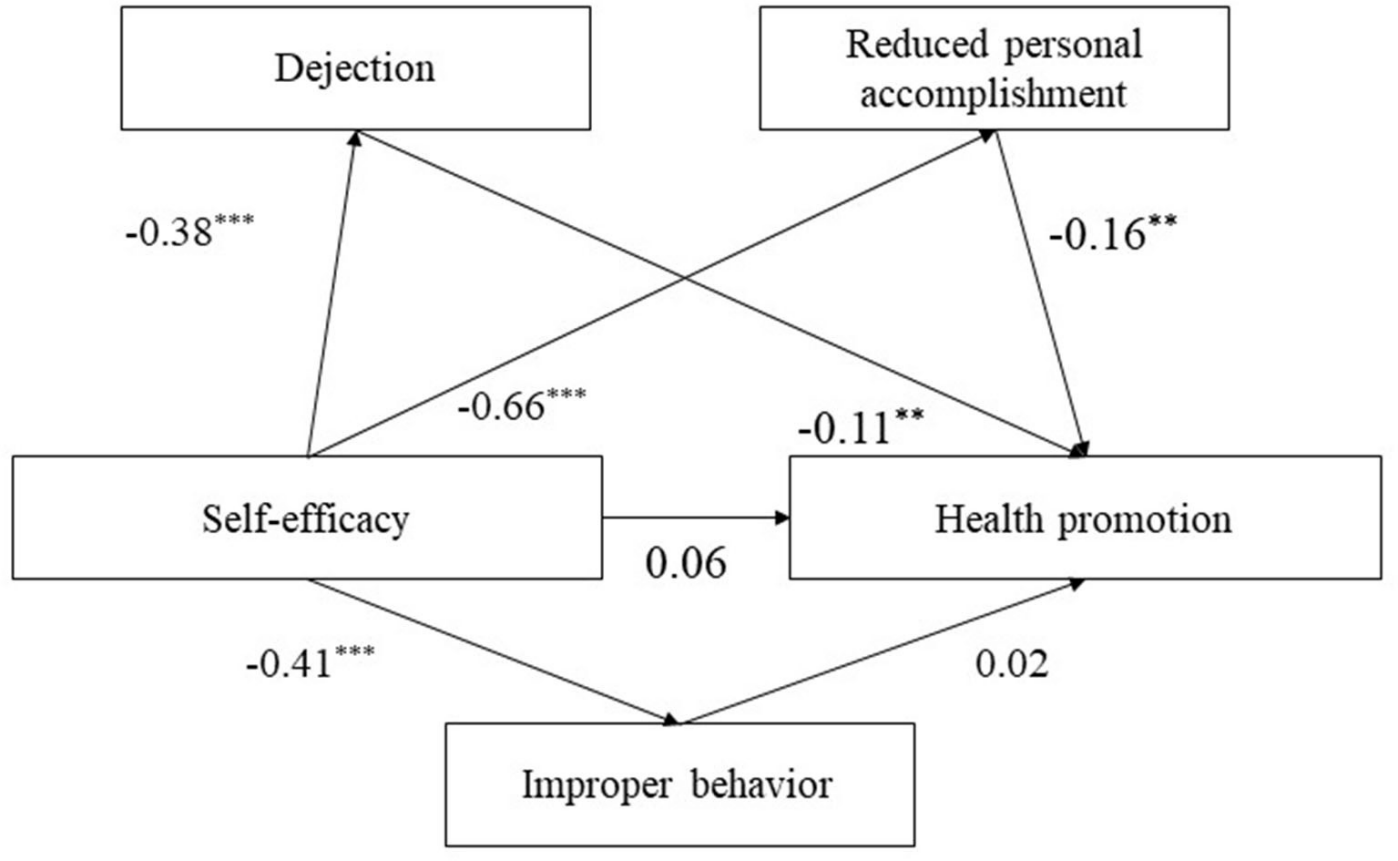
The structural model of self-efficacy, dejection, and reduced personal accomplishment on health promotion. Significant paths are presented by asterisks (**p < 0.01, ***p < 0.001).

Self-efficacy had a significant direct effect on dejection (β = −0.38, *t* = −12.75, *P* < 0.001), improper behavior (β = −0.41, *t* = −12.73, *P* < 0.001), and reduced personal accomplishment (β = −0.66, *t* = −33.13, *P* < 0.001) but no effect on health promotion (see Figure 4). Dejection (β = −0.11, *t* = −2.95, *P* < 0.01) and reduced personal accomplishment (β = −0.16, *t* = −3.44, *P* < 0.01) had a direct effect on health promotion. Self-efficacy showed a significant indirect effect on health promotion through the mediation of dejection (β = 0.04, *t* = 2.76, *P* < 0.01, 95% CI [0.01, 0.08]) and reduced personal accomplishment (β = 0.10, *t* = 3.39, *P* < 0.01, 95% CI [0.05, 0.16]). The total effect of this mediation model was significant (β = 0.20, *t* = 7.87, *P* < 0.001, 95% CI [0.15, 0.25]).

## Discussion

The present study analyzed the mechanisms underlying the association between self-efficacy and nurse–patient communication tendency through learning burnout among nursing students during the COVID-19 pandemic. Specifically, nurse–patient communication tendency was examined through three key dimensions: caring, sharing, and health promotion.

The overall descriptive results showed that nursing students were more likely to demonstrate PCC through caring and health promotion but not through sharing. These results are consistent with previous studies showing that sharing is often lost in communication with patients and families (35). One explanation for these results is that sharing complex and professional medical information with patients in a short period is difficult. When examining the nursing curriculum, the emphasis on nursing, caring skills, and health promotion suggestions in various courses is noticeable. However, how nursing students should share their medical knowledge using simple language, let alone using it during public health emergencies such as the COVID-19 pandemic, is not emphasized. Another explanation is that our data collection was conducted in February 2020, when the COVID-19 pandemic had just started. At this time, the public had little knowledge about the virus and medical care supporting the recovery, and nursing students' perspective on sharing such knowledge in patient-centered communication tendency may have been compromised.

Our initial research objective was to investigate whether self-efficacy positively predicts nurse–patient communication tendencies among nursing students. Our results demonstrated that self-efficacy is negatively associated with students' recognition of the need for caring and sharing medical information with patients in nursing, thus contradicting our hypothesis and some previous literature. The higher their levels of self-efficacy, the less likely they are to endorse warmth and support, share medical information, or validate patients' rights in the nurse–patient relationship. Nonetheless, previous studies have suggested that the self-efficacy of intern nurses is positively correlated with their clinical communication skills (38). When they received adequate self-efficacy training, intern nurses scored higher on service satisfaction (18). These unexpected results may be due to the unspecified content of self-efficacy. In our study, we measured nursing students' general self-efficacy, whereas another study [i.e., (18)] targeted a specific situation. In Shang et al. (18), their self-efficacy training was based on a nurse–patient role reversal to allow nursing students to understand patients' pain during medical processes better and improve their communication and health promotion. In this case, high self-efficacy represents higher sensitivity and involvement in communication. In our study, when asked to rate their general self-efficacy level, the nursing students may have thought only about their study abilities and biomedical knowledge concerning diagnosis and treatment, ignoring the psychosocial factors of communication. Additionally, the COVID-19 outbreak inserts all medical workers in high-risk work environments, and they shoulder the responsibility to fight the disease (39). Under these special circumstances, nursing students with higher self-efficacy might be more inspired to favor the patriarchal and protective approach in nurse–patient communication.

In support of our second hypothesis, all three aspects of learning burnout were negatively associated with self-efficacy, caring, sharing, and health promotion in PCC. The analysis of its mediation effect partially supported the third hypothesis, with only dejection and reduced personal accomplishment partially or fully mediating the three separate areas of nurse–patient communication tendency. Dejection and Reduced Self-Accomplishment Were Key in Establishing PCC.

Dejection from learning burnout partially mediated the relationships between self-efficacy and caring and between self-efficacy and sharing but fully mediated the relationship between self-efficacy and health promotion. In other words, nursing students with higher self-efficacy may have fewer negative feelings toward learning burnout and, in turn, be more inclined to provide warm and supportive PCC. This is possibly because dejection can worsen emotional well-being, leaving students less capable of caring for others. Previous studies have found that nurses' emotional states can affect their communication. In one study, an enthusiastic and optimistic mood not only improved nurses' work efficiency but also resulted in positive feedback from patients (40). The more the nurses are frustrated and lacking in interest when learning, the less willing they become to communicate with patients regarding mental and physical health, and the more they struggle to engage in PCC.

Reduced personal accomplishment partially mediated between self-efficacy and caring, while it fully mediated between self-efficacy and health promotion. Surprisingly, it did not play a role in the sharing model. A possible explanation for this result is that a low sense of self-achievement lowers self-esteem and empathy levels (41). Being confident and empathetic is crucial for establishing nurse–patient participation, wherein patients can discuss their concerns, participate in their recovery process, and obtain more health information (42). In such PCC, medical workers are more likely to alleviate patient pressure in a warm and professional manner. However, reduced personal accomplishment did not mediate between self-efficacy and sharing. One reason is that, for this sample, sharing was not as favorable as caring and health promotion in the COVID-19 situation, as discussed earlier. Another possible reason is that nursing students did not perceive the relationship between self-accomplishment and patients' status and power.

Dejection and reduced personal accomplishment played a critical mediating role between self-efficacy and nurse–patient communication tendency among the nursing students. In contrast, improper behavior did not impact any of the three models. This is probably because improper behavior may be a learning burnout outcome and manifests in different ways. In the Chinese cultural context, students often conceal certain actual behaviors of learning dissatisfaction to avoid negative consequences, such as losing attendance marks.

### Limitations and Future Direction

The present study had a few limitations that need to be addressed. First, although our investigation was conducted during the COVID-19 pandemic to see its impact on nursing students' PCC levels, we did not directly measure how the pandemic influenced students. Future studies should examine nursing students' COVID-19 perceptions and their related influences on the nursing profession. Second, the present study used a cross-sectional design; therefore, causality could not be confirmed. Future research could incorporate a longitudinal or experimental design to uncover the factors influencing PCC among nursing students further. Third, we used a Chinese modified version of the Patient–Practitioner Orientation Scale, in which the term was worded as doctor-patient communication but meant all health practitioners in general in the Chinese context. This needs to be re-worded when replicating this study in other cultural contexts. Future studies should also compare the response between nurses and doctors using the same scale. Fourth, most nursing students in our sample were women, which is representative of the women-to-men ratio in the nursing industry in China. However, we still need to be cautious while drawing conclusions about the relationship between self-efficacy, learning burnout, and PCC level in relation to gender. Finally, participants were recruited through convenience sampling by targeting nursing students in one vocational college in Fujian. During the data collection, all students stayed off-campus and were distributed across southeast China due to the pandemic control policy. Therefore, the generalizability of these results was limited. Compared to other countries, nursing students from mainland China tend to have more course hours on medical science theory and less emphasis on humanities (43). The learning burnout in the present model might be weakened in nursing students with less study workload. Future studies could consider the cross-cultural comparison in PCC learning and its related factors.

## Conclusion

The present study was the first to explore the mechanism by which self-efficacy and learning burnout influence nursing students' PCC tendency during the early stage of a public health crisis such as the COVID-19 pandemic. Self-efficacy influences nurse–patient communication through learning burnout. Specifically, dejection and reduced personal accomplishment—two aspects of learning burnout—may compromise nursing students' willingness to engage in PCC. Thus, the importance and meaningfulness of PCC, especially during critical health situations such as pandemics, should be emphasized further in future nursing education. Future nurses need to be equipped with a humanistic care mindset, respecting patients' involvement in medical treatment recovery. At the same time, medical education institutions need to note students' self-efficacy and reduce their learning burnout level (25). Students who discover their self-worth and emotional balance during their Nursing studies could become warm-hearted professionals.

## Data Availability Statement

The raw data supporting the conclusions of this article will be made available by the authors, without undue reservation.

## Ethics Statement

The studies involving human participants were reviewed and approved by Ethics Committee of Fujian Health College. The patients/participants provided their written informed consent to participate in this study.

## Author Contributions

LW designed the theoretical framework. JW analyzed the data. WS and LW collected the data. All authors wrote the manuscript.

## Funding

This study was funded by the research project career commitment and influencing factors of medical higher vocational students of the educational research project for young and middle-aged teachers and the Education Department of Fujian Province (JZ180564).

## Conflict of Interest

The authors declare that the research was conducted in the absence of any commercial or financial relationships that could be construed as a potential conflict of interest.

## Publisher's Note

All claims expressed in this article are solely those of the authors and do not necessarily represent those of their affiliated organizations, or those of the publisher, the editors and the reviewers. Any product that may be evaluated in this article, or claim that may be made by its manufacturer, is not guaranteed or endorsed by the publisher.
